# Protocol for generating nociceptor-enriched peripheral sensory neurons from human induced pluripotent stem cells

**DOI:** 10.1016/j.xpro.2026.104944

**Published:** 2026-11-04

**Authors:** Arslan Ghazanfar, James P. Koch, Thomas Wartmann, Ahmed Y. Sanin, Sebastian Samer, Muhammad Irfan Siddique, Ulrike Steinmann, Christoph Hoeschen, Roland S. Croner, Ulf D. Kahlert

**Affiliations:** 1 Molecular and Experimental Surgery, University Clinic for General-, Visceral-, and Transplant Surgery, Faculty of Medicine, Otto-Von-Guericke-University Magdeburg, 39120 Magdeburg, Germany; 2 Research Group Neuroplasticity, Leibniz Institute for Neurobiology, 39118 Magdeburg, Germany; 3 Department of Pharmaceutics, Faculty of Pharmacy, Northern Border University, Rafha, Saudi Arabia; 4 Institute of Automation Technology, Otto-von-Guericke University, Universitätsplatz 2, 39106 Magdeburg, Germany; 5 Institute of Medical Technology, Faculty of Electrical Engineering and Information Technology, Otto-von-Guericke University, Universitätsplatz 2, 39106 Magdeburg, Germany; 6 Research Campus Stimulate, Otto-Hahn-Str. 2 G82-173, 39106 Magdeburg, Germany; 7 Insitute for Quality Insurance in Operative Medicine, Magdeburg, Germany

**Keywords:** Cell culture, Cell isolation, Gene Expression, Stem Cells, Cell Differentiation

## Abstract

Human induced pluripotent stem cells (hiPSCs) are a valuable system for studying human development, modeling disease-associated cellular phenotypes, and generating difficult-to-access cell types, such as sensory neurons (SNs). Here, we present a stepwise differentiation protocol for generating peripheral SNs from hiPSCs under defined culture conditions. We describe steps for expansion of hiPSCs, induction of neural crest cells (NCCs), dissociation and replating of NCCs, and maturation into SNs. This protocol yields nociceptor-enriched SN cultures.

## Highlights

• Protocol for stepwise differentiation of human iPSCs into sensory neurons
• Steps for replating neural crest cells for sensory neuron induction
• Instructions for quality control using microscopy and molecular assays
• Determine nociceptor-like features by substance P release

## Abstract (editor highlights)

Publisher’s note: Undertaking any experimental protocol requires adherence to local institutional guidelines for laboratory safety and ethics.

## Graphical abstract

**Figure:**
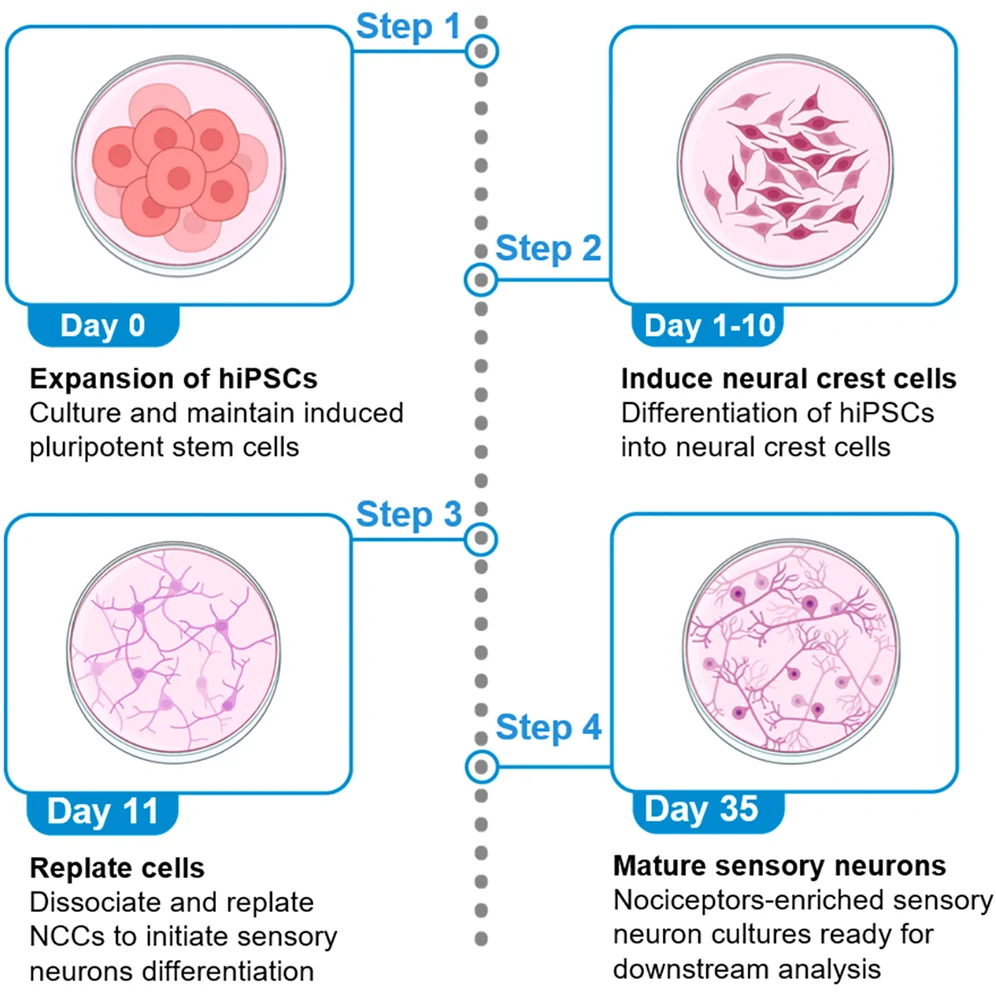

## Before you begin

The protocol describes a method for differentiating sensory neurons (SNs) from human induced pluripotent stem cells (hiPSCs), including the iPS12 and iMR90-4 lines (Figure 1A). During this process, the cells undergo progressive developmental transitions that lead to the generation of a nociceptor-enriched sensory neuron culture. Sensory neurons produced using this protocol can be maintained in culture for extended periods, including beyond 8 weeks when required. All procedures described in this protocol are performed in a laminar flow hood using appropriate sterile and aseptic techniques.

**Figure 1:**
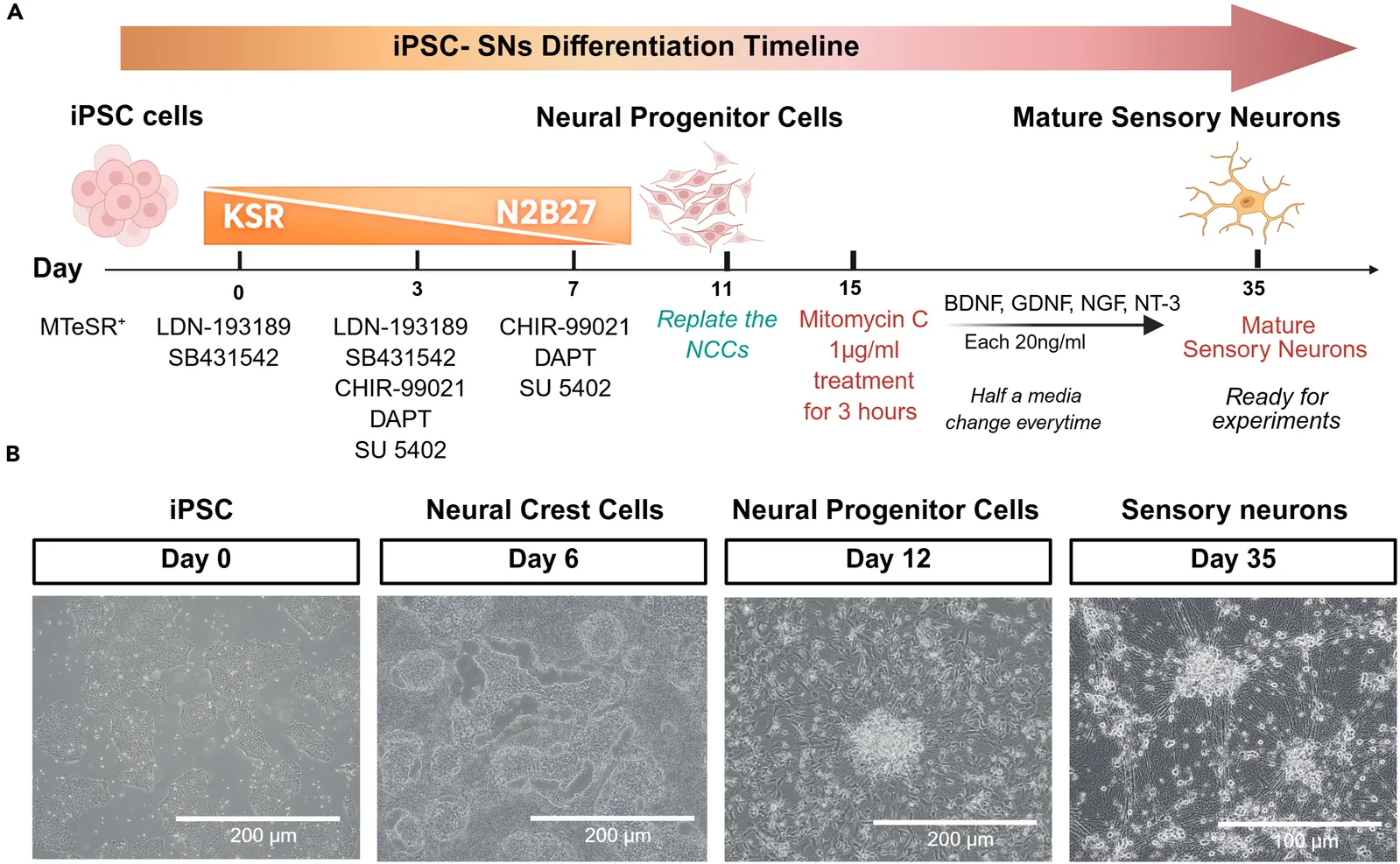
Differentiation workflow of iPSC-derived sensory neurons (A) Schematic representation of the sensory neuron (SN) differentiation timeline showing the major developmental stages (top) and the growth factors or media conditions applied at each stage of differentiation (bottom). (B) Representative phase contrast images showing the morphological progression of the differentiation process, including iPSCs at day 0, neural crest cells (NCCs) at day 6, neural crest progenitors (NCPs) at day 12, and mature sensory neurons at day 35.

The workflow consists of four main stages: (1) expansion of hiPSCs, (2) differentiation into neural crest cells (NCCs), (3) dissociation and replating of NCCs, and (4) differentiation into sensory neurons (SNs) (Figure 1B). The iPSC-SNs are reseeded on day 11 according to the protocol^1^ to generate a dorsal root ganglion (DRG) like nociceptor enriched SNs composition.

This protocol is designed for NCC differentiation in 6-well plates. The iPSC-12 and iMR90-4 iPSC lines are differentiated in 6-well plates during the dual SMAD inhibition phase^1^^,^^2^^,^^3^ to generate NCCs (Figure 1A). NCCs are subsequently reseeded into 12-well or 24-well plates for sensory neuron (SN) differentiation, depending on the experimental design. Reagent volumes should be adjusted proportionally when scaling the protocol to multiple plates or other culture formats.

### Innovation

This protocol presents an optimized workflow for generating human hiPSC-derived sensory neurons through a neural crest cell intermediate, with improvements in differentiation efficiency, culture purity, and experimental usability. A key advancement of this protocol is the demonstration that a high yield of neural crest cells (NCCs) can be obtained by day 11 of differentiation, as confirmed by characteristic NCC morphology and increased expression of NCC-associated markers. This provides a reliable time point for replating cells into sensory neuron differentiation conditions.

Another important optimization is the timing of Mitomycin C treatment. Because cells remain stressed after replating, treatment is performed at day 15 rather than earlier, with a controlled 2-h exposure. This reduces proliferating non-neuronal cells while minimizing Mitomycin C-associated cell death and stress, resulting in a more enriched neuronal culture.

This protocol also defines a practical experimental window for downstream applications. qPCR analysis confirms that neuronal and nociceptor-associated markers are already detectable at 35 days in vitro (DIV35), indicating that sensory neurons can be used for experiments from this stage onward. In addition, the cultures can be maintained beyond 8 weeks, allowing both medium-term and long-term studies of sensory neuron maturation and function. Together, these refinements make the protocol reproducible, practical, and suitable for generating stable sensory neuron cultures for molecular, imaging, and functional assays.

### Preparation of Matrigel-coated plates for iPSC expansion and NCC differentiation

**Timing: 1.5–2.5 h**

1. In a laminar flow hood, place a sterile 15 mL conical tube together with a bottle of cold, sterile DMEM/F-12 medium.
2. Add 11 mL of cold DMEM/F-12 medium to the conical tube.
3. Retrieve a 1.5 mg aliquot of Matrigel from the freezer and immediately add 1 mL of cold DMEM/F-12 medium using a micropipette.
4. Gently pipette the mixture up and down until the Matrigel is completely dissolved.
5. Transfer the dissolved Matrigel solution immediately into the 15 mL conical tube containing the prechilled DMEM/F-12 medium.
6. Mix the solution gently using a 10 mL pipette. Avoid vigorous pipetting, as bubble formation may interfere with even coating of the culture surface.
7. Add 1 mL of the diluted Matrigel solution to each well of a 6-well plate.
8. Allow to set for one to two hours in a 37°C incubator.
9. If the coated plates are not used immediately, seal them with parafilm and store at 4°C.
  a. Before use, warm the plates either at room temperature for 1 h or at 37°C for 1 h in an incubator.

### Preparation of laminin/poly-L-ornithine-coated plates for SN differentiation

**Timing: 2.5–3.5 h**

10. Gently mix the ready-to-use laminin and poly-L-ornithine solution several times to ensure a homogeneous coating solution before use.
11. Add sufficient coating solution to fully cover the culture surface, using approximately 0.2–0.25 mL/cm^2^.
12. Incubate the culture vessel at room temperature for 2 to 3 h.
13. Carefully aspirate the coating solution and wash the surface three times with sterile PBS before plating the cells.
14. The coated plates may be used immediately or stored at 4°C for up to 1 week. For storage, keep the plates filled with sterile PBS and seal them with parafilm.
15. Remove the PBS only immediately before cell seeding, and do not allow the coated surface to dry completely.

**CRITICAL:** Sensory neurons (SNs) show improved growth and maintenance on laminin/poly-L- ornithine-coated plates, which also helps minimize the presence of non-neuronal cell types in the culture. The culture should be handled with particular care, as sensory neurons are delicate and do not adhere strongly to the laminin/poly-L-ornithine-coated surface.

## Key resources table

**Table:** 

REAGENT or RESOURCE	SOURCE	IDENTIFIER
**Antibodies**		

anti-PAX6, mouse (dilution 1:1000)	Abcam	Cat#ab78545
Purified anti-Nestin, mouse (dilution 1:1000)	BioLegend	Cat#656801
anti-MAP2, mouse (dilution 1:1000)	BioLegend	Cat#11442
Islet 1, Rabbit (dilution 1:500)	Proteintech	Cat#83784-4-RR
Peripherin (F3J1R), rabbit (dilution 1:1000)	Cell Signaling	Cat#85624T
TRPV1, rabbit (dilution 1:500)	Abcam	Cat#ab3487
Alexa Fluor 647 anti-rabbit (dilution 1:1000)	ThermoFisher Scientific	Cat# A-21245
Alexa Fluor 488, anti-mouse (dilution 1:1000)	ThermoFisher Scientific	Cat#A32723TR

**Chemicals, peptides, and recombinant proteins**		

mTeSR Plus	STEMCELL Technologies	Cat#100–0276
KnockOut DMEM Medium	Gibco	Cat#10829018
KnockOut Serum Replacement Medium	Gibco	Cat#10828028
DMEM/F-12 with 15 mM HEPES	STEMCELL Technologies	Cat#36254
Neurobasal Medium	Gibco	Cat#21103049
Dulbecco’s phosphate-buffered saline (DPBS)	Thermo Fisher Scientific	Cat#14190144
GlutaMAX Supplement 100x	Gibco	Cat#35050061
MEM NEAA 100x	Gibco	Cat#11140050
Penicillin-Streptomycin (10,000 U/mL)	Gibco	Cat#15140122
N2 Supplement 100x	Capricorn Scientific	Cat#N2-K
NCS21 Neuronal Supplement (B27) 50x	Capricorn Scientific	Cat#C21-H
2-Mercaptoethanol (50 mM)	Gibco	Cat#31350010
Accutase	BioLegend	Cat#423201
Matrigel	Corning	Cat#CLS354277
Vitronectin XF	STEMCELL Technologies	Cat#07180
Laminin/Poly-L-Ornithine Coating Solution	Merck	Cat#LPLO001
Bambanker	NIPPON Genetics	Cat#CS-07–001/BBH01
Y-27632	MedChemExpress	Cat#HY-10583
LDN-193189	Tocris	Cat#6053
SB431542	Tocris	Cat#1614
CHIR-99021	Tocris	Cat#4423
DAPT	MedChemExpress	Cat#HY-13027
SU 5402	MedChemExpress	Cat#HY-10407
BDNF	BioLegend	Cat#788904
GDNF	BioLegend	Cat#798404
Beta-NGF	MedChemExpress	Cat#HY-P73316
NT-3	ABclonal	Cat#RP00496
Dimethylsulfoxide (DMSO)	Merck	Cat#D8418
Mitomycin C	Gerbu Biotechnik	Cat#1920
Hoechst 20 mM	Thermo Fisher Scientific	Cat#62249
LIVE/DEAD™ Cell Imaging Kit (488/570)	Thermo Fisher Scientific	Cat#R37601
Roti Histofix 4% Paraformaldehyde (PFA)	Carl Roth	Cat#P087.1
Triton X-100	Sigma Aldrich	Cat#T8787
Bovine serum albumin (BSA) 25%	Gibco	Cat#A10008-01
Invitrogen UltraPure, DNase/RNase-free distilled water	Fisher Scientific	Cat#11538646

**Critical commercial assays**		

Luna Script RT SuperMix Kit	Biolabs	Cat#M3010L
Luna Universal qPCR Master Mix	Biolabs	Cat#3003S
ReliaPrep miRNA Cell and Tissue Miniprep System	Promega	Cat#Z6212
Human Substance P Elisa Kit	AFG Bioscience	Cat#EK711438

**Experimental models: cell lines**		

Human iPSC Line (Episomal, MSC) iPS12	ALSTEM	Cat#iPS12
iPS(IMR90)-4	WiCell	Cat#WiSCi004-B

**Oligonucleotides**		

RT-qPCR Primer: *NANOG* F CCTCCATGGATCTGCTTATT	Microsynth AG	Cat#5670592
RT-qPCR Primer: *NANOG* R ATCTGCTGGAGGCTGAGGTA	Microsynth AG	Cat#5670593
RT-qPCR Primer: *POU5F1* F AGAAAGCGAACCAGTATCGAGAA	Microsynth AG	Cat#5670596
RT-qPCR Primer: *POU5F1* R CTCAAAATCCTCTCGTTGTGCAT	Microsynth AG	Cat#5670597
RT-qPCR Primer: *TRPV1* F GCACAGGAGAGCAAGAACATC	Microsynth AG	Cat#5670598
RT-qPCR Primer: *TRPV1* R GTCCAGTTCACCTCGTCCAC	Microsynth AG	Cat#5670599
RT-qPCR Primer: *RUNX1* F TGAGCTGAGAAATGCTACCGC	Microsynth AG	Cat#4552615
RT-qPCR Primer: *RUNX1* R ACTTCGACCGACAAACCTGAG	Microsynth AG	Cat#4552616
RT-qPCR Primer: *ISL1* F GCAGCAGCCCAATGACAAAAC	Microsynth AG	Cat#4552621
RT-qPCR Primer: *ISL1* R TGCCAGTGGAATTAGAGCCC	Microsynth AG	Cat#4552622
RT-qPCR Primer: *PAX6* F GCATGCAGAACAGTCACAGCGGAG	Microsynth AG	Cat#4640943
RT-qPCR Primer: *PAX6* R CTGTTGCTTTTCGCTAGCCAGGTT	Microsynth AG	Cat#4640944
RT-qPCR Primer: *SOX10* F ATGAACGCCTTCATGGTGTGGG	Microsynth AG	Cat#5974052
RT-qPCR Primer: *SOX10* R CGCTTGTCACTTTCGTTCAGCAG	Microsynth AG	Cat#5974053
RT-qPCR Primer: *MAP2* F GCAGTTCTCAAAGGCTAGAC	Microsynth AG	Cat#4640945
RT-qPCR Primer: *MAP2* R TTGATCGTGGAACTCCATCT	Microsynth AG	Cat#4640946
RT-qPCR Primer: *NESTIN* F GCTGAAGCCCTGGGGAAAGT	Microsynth AG	Cat#4640941
RT-qPCR Primer: *NESTIN* R CCAGGGGAGTGGAGTCTGGA	Microsynth AG	Cat#4640942
RT-qPCR Primer: *ACTIN* F ACC CCA GCC ATG TAC GTT	Microsynth AG	Cat#4552619
RT-qPCR Primer: *ACTIN* R GGT GAG GAT CTT CAT GAG GTA G-	Microsynth AG	Cat#4552620

**Software and algorithms**		

Bio-Rad CFX Maestro	BioRad	Cat#120133758
Graphpad Prism 10	Graphpad Software, USA	https://www.graphpad.com/
Zotero	Zotero	https://www.zotero.org/
Fiji	ImageJ	https://imagej.net/imagej-wiki-static/Citing

**Other**		

Cell culture plate, 6 well	SARSTEDT	Cat#833920
Cell culture plate, 12 well	SARSTEDT	Cat#833921
Cell culture plate, 24 well	SARSTEDT	Cat#833922
Tissue culture cover slips 13mm	SARSTEDT	Cat#83.1840.002
Sigma laboratory centrifuge	Sigma	Cat#92644
Zeiss Axio Osberver 7	Zeiss	Cat#431007–9904–000
Herasafe 2012.1.2 Biosafety cabinet	ThermoFisher Scientific	Cat#42905778
Heracell Vios 250i CO_2_ incubator	ThermoFisher Scientific	Cat#30645070
C1000 Touch Thermocycler	Bio-Rad	Cat#785BR19946
Thermocycler	Biometra Trio 48	Cat#846-X-070–723

## Materials and equipment

**Table:** Preparation, Working Concentrations, and Storage of Reagents

Reagent	Stock concentration	Working concentration	Diluent	Storage
LDN-193189	10 mM	100 nM	DMSO	−20^o^C
SB431542	10 mM	10 μM	DMSO	−20^o^C
CHIR-99021	10 mM	3 μM	DMSO	−20^o^C
DAPT	10 mM	10 μM	DMSO	−20^o^C
SU 5402	10 mM	10 μM	DMSO	−20^o^C
BDNF	20 μg/mL	20 ng/mL	DPBS	−20^o^C
GDNF	20 μg/mL	20 ng/mL	DPBS	−20^o^C
Beta-NGF	20 μg/mL	20 ng/mL	DPBS	−20^o^C
NT-3	20 μg/mL	20 ng/mL	Sterile distilled water	−20^o^C
Y-27632	10 mM	10 μM	DMSO	−20^o^C
Mitomycin C	1 mg/mL	1 μg/mL	Sterile distilled water	−20^o^C

**Table:** NCC differentiation medium (KSR medium)

Reagent	Storage	Final concentration	Amount
KnockOut DMEM Medium	4°C	N/A	38.5 mL
KnockOut Serum Replacement Medium	4°C	N/A	10 mL
GlutaMAX Supplement	4°C	1x	500 μL
MEM NEAA	4°C	1x	500 μL
Penicillin-Streptomycin (10,000 U/mL)	4°C	1x	500 μL
**Total**	–	**N/A**	**50 mL**

Store at 4°C. Fresh preparation is recommended; however, the solution can be stored for up to 10 days.

**Table:** Volume of small molecules used in NCCs differentiation

Reagent	Final concentration	Amount
LDN-193189	100 nM	50 μL
SB431542	10 μM	50 μL
CHIR-99021	3 μM	50 μL
DAPT	10 μM	50 μL
SU 5402	10 μM	50 μL

***Note:*** The volumes of small molecules listed in the table correspond to the amounts required to prepare 50 mL of NCC differentiation medium.

**CRITICAL:** Small-molecule reagents should always be added freshly to a small aliquot of medium at the time of feeding. Avoid adding these reagents to bulk volumes of medium, as they remain stable for only a limited period after preparation.

**CRITICAL:** LDN-193189 is unstable and light-sensitive. Prepare small-volume aliquots for example (30 μL), and protect them from light during storage and handling and add freshly at each feeding. Avoid repeated freeze-thaw cycles for all small-molecule reagents.

***Note:*** The medium should not be warmed before use. Instead, place it at room temperature for 1 h prior to use.

**Table:** SNs Differentiation media (N2B27 media)

Reagent	Storage	Final concentration	Amount
N2 Supplement 100x	−20^o^C	1x	500 μL
NCS21 Neuronal Supplement (B27) 50x	−20^o^C	1x	1000 μL
Neurobasal Medium	4°C	N/A	37.5 mL
GlutaMAX Supplement	4°C	1x	500 μL
Penicillin-Streptomycin (10,000 U/mL)	4°C	1x	500 μL
2-Mercaptoethanol (50 mM)	4°C	0.01mM	10 μL
**Total**	–	**N/A**	**50 mL**

Store at 4°C. Fresh preparation is recommended; however, the solution can be stored for up to 14 days.

**Table:** Volume of small molecules reagents used in SNs differentiation

Reagent	Final concentration	Amount
BDNF	20 ng/mL	50 μL
GDNF	20 ng/mL	50 μL
Beta-NGF	20 ng/mL	50 μL
NT-3	20 ng/mL	50 μL

***Note:*** The volumes of small molecules listed in the table correspond to the amounts required to prepare 50 mL of NCC differentiation medium.

**CRITICAL:** Small-molecule reagents should always be added freshly to a small aliquot of medium at the time of feeding. Avoid adding these reagents to bulk volumes of medium, as they remain stable for only a limited period after preparation.

***Note:*** The medium should not be warmed before use. Instead, place it at room temperature for 1 h prior to use.

**Table:** Antibodies to be used

Antibody	Storage	Host species	Working dilution
Anti-MAP2	4°C	Mouse	1:500
Peripherin	−20^o^C	Mouse	1:500
Anti-Islet 1	−20^o^C	Rabbit	1:500
Anti-TRPV1	−20^o^C	Rabbit	1:500
Anti-PAX6	−20^o^C	Mouse	1:500
Anti-Nestin	4°C	Mouse	1:1000
Alexa Fluor 647	4°C	Goat	1:1000
Alexa Fluor 488	4°C	Goat	1:1000

**Table:** RT-qPCR: Preparation of the RT-qPCR master mix for a 20 μL reaction

Reagent	Stock concentration	Final concentration	Volume
Luna Universal qPCR Master Mix	2x	1x	10 μL
Forward primer	10 μM	0.25 μM	0.5 μL
Reverse primer	10 μM	0.25 μM	0.5 μL
Template DNA	Variable	< 100 ng	5 μL
Nuclease-free water			4 μL
Luna Universal qPCR Master Mix	2x	1x	10 μL
**Total**	–	–	**20 μL**

**CRITICAL:** Prior to reverse transcription (RT) reaction, the RNA concentration of each sample is measured, and the volume corresponding to 1 μg is used for each sample. An equal total RNA mass of 1 μg is used for each RT reaction, while the RNA sample volume is adjusted according to measured concentration. Nuclease-free water is added to maintain a consistent final reaction volume across all samples. RT is then performed to generate cDNA. Subsequently, the qPCR master mix is prepared at 4^o^C and thoroughly mixed. The required volume master mix is dispensed into the wells of qPCR plate, followed by the appropriate amount of cDNA. The qPCR plate is centrifuged at 300 × *g* for 1 min to ensure the uniform distribution and mixing of the reaction components before running the thermocycler program.

**Table:** Real-time qPCR thermocycler program protocol

Cycle step	Temperature	Time	Cycles
Initial Denaturation	95°C	60 s	1
Denaturation	95°C	15 s	40–45
Extension	60°C	30 s	40–45
Melt Curve	60–95°C	Various	1

## Step-by-step method details

### Induced pluripotent stem cell expansion

**Timing: 5 days**

This step recovers cryopreserved iPSCs and seeds them onto Matrigel-coated 6-well plates in mTeSR Plus medium supplemented with Y-27632. The goal is to promote high post-thaw survival, uniform cell distribution, and healthy colony formation before further expansion.

1. Prepare Matrigel-coated 6-well plates and incubate them at 37°C for at least 1 h before use.
2. Prepare mTeSR Plus medium supplemented with Y-27632 at a 1:1000 dilution.
3. Transfer the cryovial directly from dry ice to a 37°C water bath.
  a. Gently swirl the cryovial until only a small ice crystal remains.
  b. Submerge only the lower half of the cryovial in the water bath.

**CRITICAL:** Do not fully immerse the cryovial in water. Keep the cap above the water level to reduce the risk of contamination.

**CRITICAL:** Do not warm mTeSR Plus medium in a hot water bath. Allow the medium to reach room temperature before use to prevent degradation of heat-sensitive growth factors, especially FGF2.

4. Disinfect the outside of the cryovial with ethanol before placing it in the biosafety cabinet.
5. Gently resuspend the thawed cells using a 1000 μL pipette.
6. Slowly transfer the cell suspension into a 15 mL conical tube containing 5 mL mTeSR Plus medium.
  a. If a small volume of cell suspension remains in the cryovial, add approximately 200 μL of the diluted cell suspension back into the vial.
  b. Gently resuspend the remaining cells and transfer them into the same conical tube.

**CRITICAL:** Add the thawed cells slowly and gently over several minutes to reduce osmotic shock and improve cell survival.

7. Centrifuge the cells at 300 × *g* for 5 min.

***Note:*** All centrifugation steps are performed with these parameters at RT.

8. During centrifugation, aspirate Matrigel from the coated 6-well plate.
9. Add 1 mL mTeSR Plus medium supplemented with Y-27632 at a 1:1000 dilution to each well.
10. After centrifugation, carefully aspirate the supernatant without disturbing the cell pellet.
11. Resuspend the cell pellet in an appropriate volume of mTeSR Plus medium supplemented with Y-27632 at a 1:1000 dilution.
12. Plate 1 mL cell suspension into each well of the prepared 6-well plate.
13. Examine the cells under a microscope to confirm even distribution across the well.
14. Place the plate in a 37°C incubator under hypoxic conditions.
15. Gently rock the plate back and forth to promote uniform cell distribution.

**CRITICAL:** It is important to refresh the mTeSR medium after 24 h to remove Rock inhibitor. Thereafter, replace the medium daily with 2 mL of fresh mTeSR medium per well during iPSC stage.

**CRITICAL:** Healthy iPSC colonies should show clearly defined, bright edges with minimal spontaneous differentiation when assessed by phase contrast microscopy (Figure 1B).

### Differentiation of iPSCs into NCCs

**Timing: 11 days**

This step induces human iPSCs toward neural crest cells (NCCs) with sensory neuron fate using staged small-molecule supplementation through dual SMAD inhibition^4^ and a gradual transition from KSR medium to N2B27 medium. NCC aggregates usually begin to appear around day 4, become denser and darker over time, and are typically clearly visible by day 8 under a phase contrast microscope (Figures 2A–2F).

16. Use healthy iPSC cultures at approximately 80% confluency with minimal spontaneous differentiation.
17. Prepare Matrigel coated plates and all required media before starting the differentiation workflow.
18. Aspirate mTeSR Plus medium from undifferentiated iPSCs and wash the cells once with DPBS.
19. Add 1 mL Accutase to each well and incubate the cells for 10 min at 37°C.
20. Check and confirm the cells dissociation under a microscope.
  a. Proceed when the colonies dissociate into single cells.
  b. Check that the cell suspension appears cloudy.
21. Add 2 volumes of mTeSR Plus medium to stop the Accutase reaction.
22. Transfer the cell suspension to a 50 mL conical tube.
23. Centrifuge the cells at 200 × *g* for 5 min.
24. Resuspend the cell pellet in mTeSR Plus medium supplemented with Y-27632 at a 1:1000 dilution.
25. Add 2 mL mTeSR Plus medium supplemented with Y-27632 at a 1:1000 dilution to each well.
26. Count the cells using hemocytometer.
  a. Mix 20 μL of cell suspension into 20 μL of Trypan blue.
  b. Mix gently by pipetting, then load 10 μL of the cell suspension into the counting chamber of a hemocytometer.
  c. Count the cells.
27. Aspirate the Matrigel coating solution.
28. Seed the iPSCs at 40,000 cells/cm^2^ into each coated well.
29. Allow the cells to recover and expand until the culture reaches approximately 70% confluency (Figure 2A).

**CRITICAL:** Start differentiation only when the iPSC culture reaches approximately 70% confluency. Overconfluent or unhealthy cultures may reduce NCC induction efficiency.

***Note:*** The detailed compositions of KSR medium and N2B27 medium are listed in the key resources table.

**CRITICAL:** Replace the differentiation medium daily from day 0 until day 11.

30. On day 0, completely remove the iPSC maintenance medium and replace it with 100% KSR differentiation medium supplemented with 100 nM LDN193189 and 10 μM SB431542.

***Note:*** LDN193189 and SB431542 inhibit the two major branches of SMAD signaling and promote neural ectodermal specification (Figure 2A, DIV3).

31. On day 1, maintain the cells in 100% KSR differentiation medium supplemented with 100 nM LDN193189 and 10 μM SB431542.
  a. Completely remove the existing medium.
  b. Replace it with freshly prepared differentiation medium containing both inhibitors.

***Note:*** Daily replacement with freshly prepared medium maintains continuous inhibition of SMAD signaling.

32. On day 2, completely replace the medium with fresh 100% KSR differentiation medium supplemented with 100 nM LDN193189 and 10 μM SB431542.

***Note:*** Continuous inhibition of BMP and TGF-β/Activin/Nodal signaling supports the progressive loss of pluripotency and commitment toward the neuroectodermal lineage.

33. On day 3, replace the medium with fresh 100% KSR differentiation medium supplemented with 100 nM LDN193189 and 10 μM SB431542. Maintain the cells under these conditions for another 24 h.

***Note:*** Maintain the cells entirely in KSR differentiation medium from days 0–3. Begin the gradual transition toward N2B27 medium on day 4.

34. On day 4, initiate the gradual transition from KSR differentiation medium to N2B27 medium.
  a. Prepare a medium mixture containing 75% KSR differentiation medium and 25% N2B27 medium.
  b. Supplement the medium with 100 nM LDN193189, 10 μM SB431542, 3 μM CHIR99021, 10 μM DAPT, and 10 μM SU5402.
  c. Completely replace the existing medium with the freshly prepared medium mixture.
35. On day 5, maintain the cells in 75% KSR differentiation medium and 25% N2B27 medium supplemented with 100 nM LDN193189, 10 μM SB431542, 3 μM CHIR99021, 10 μM DAPT, and 10 μM SU5402.
  a. Replace the existing medium with freshly prepared medium of the same composition.
  b. Monitor the culture morphology during the transition period.

***Note:*** The gradual change in medium composition allows the cells to adapt to increasingly neural-supportive culture conditions without an abrupt change in culture medium (Figure 2, DIV6).

36. On day 6, increase the proportion of N2B27 medium.
  a. Prepare a medium mixture containing 50% KSR differentiation medium and 50% N2B27 medium.
  b. Supplement the medium with 100 nM LDN193189, 10 μM SB431542, 3 μM CHIR99021, 10 μM DAPT, and 10 μM SU5402.
  c. Completely replace the existing medium with the freshly prepared medium mixture.
37. On day 7, maintain the cells in 50% KSR differentiation medium and 50% N2B27 medium.
  a. Discontinue LDN193189 and SB431542 from this point onward.
  b. Supplement the medium with 3 μM CHIR99021, 10 μM DAPT, and 10 μM SU5402 only.
  c. Continue using these three small molecules for the remaining of the differentiation period.

**CRITICAL:** Do not add LDN193189 or SB431542 from day 7 onward.

38. On days 8 and 9, further shift the culture toward N2B27-based neural differentiation conditions.
  a. Prepare a medium mixture containing 25% KSR differentiation medium and 75% N2B27 medium.
  b. Supplement the medium with 3 μM CHIR99021, 10 μM DAPT, and 10 μM SU5402.
  c. Completely replace the existing medium with freshly prepared medium of the same composition on each day.
  d. Monitor the culture for the appearance of aggregate- or ridge-like structures.

***Note:*** By day 9, replace 75% of the KSR differentiation medium with N2B27 medium, supporting the establishment of a neural progenitor phenotype. Clear aggregate or ridge-like structures are typically visible by day 8 (Figure 2, DIV8).

39. On day 10, complete the transition from KSR differentiation medium to N2B27 medium.
  a. Completely remove the KSR-containing medium.
  b. Replace it with 100% N2B27 medium supplemented with 3 μM CHIR99021, 10 μM DAPT, and 10 μM SU5402.
40. On day 11, maintain the cells in 100% N2B27 medium supplemented with 3 μM CHIR99021, 10 μM DAPT, and 10 μM SU5402.

***Note:*** At this stage, the neural induction period is complete, and the resulting neural progenitor population is ready for subsequent neural lineage specification or neuronal differentiation. The cells can subsequently be dissociated and replated onto an appropriate extracellular matrix-coated surface for further differentiation and maturation (Figure 2, DIV11).

**CRITICAL:** If clear NCC ridge formation is not observed (Figure 2B), it is recommended to terminate the differentiation, as the sensory neuron yield is likely to be low and/or the level of contaminating cells may be too high for reliable downstream analysis. See troubleshooting problem 1.

***Note:*** It is important to perform a parallel differentiation up to the NCC stage for quality control (QC) purposes. This allows RNA isolation or immunofluorescence analysis on day 8 or day 10 using a plate that is treated in parallel with the main differentiation plate.

**CRITICAL:** Do not wash the cells with cold DPBS.

**CRITICAL:** Always take pictures on every feeding day to properly follow progression.

**Figure 2:**
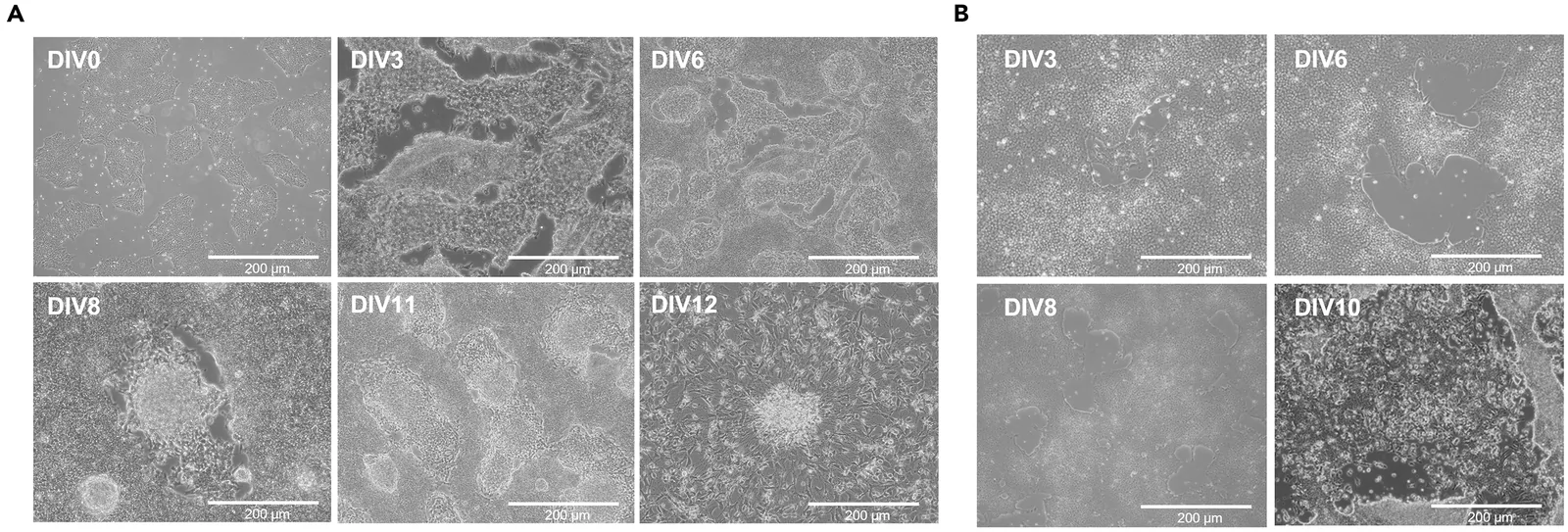
Morphological progression of neural crest cell (NCC) differentiation from iPSCs Representative phase contrast images showing the sequential morphological changes observed during NCC differentiation at the indicated time points: (A) Cultures show progressive transition from iPSC colonies to NCC-like morphology, with the appearance of dense cell aggregates at intermediate stages and expanded migratory cells following replating. (B) Representative phase contrast images showing the morphology of improperly differentiated NCC cultures at the indicated time points. Scale bars, 200 μM.

### Differentiation of NCCs to SNs (nociceptor enrichment)

**Timing: 30 days**

This step differentiates neural crest cells (NCCs) into sensory neurons (SNs) after replating on day 11 of NCC differentiation. During maturation, the cells progressively form aggregates and ganglion-like structures, and the differentiated sensory neurons can be maintained for more than 7 weeks under these culture conditions.

41. Prepare laminin- and poly-L-ornithine-coated plates and incubate them at room temperature for at least 2 h before use.
42. On day 11 of NCC differentiation, aspirate the culture medium and wash the cells once with DPBS.
43. Add 1 mL Accutase to each well and incubate the cells at 37°C for 10 min, or until the cells begin to detach.

**CRITICAL:** If the cells do not detach within 10 min, extend the incubation and allow the cells to detach spontaneously. Avoid excessive pipetting up and down to generate single-cell suspensions, as this may damage cell morphology and reduce cell viability after replating. See the troubleshooting problem 2.

44. Transfer the detached cell suspension into a 50 mL conical tube using a 1000 μL pipette.
45. Rinse the 6-well plate with N2B27 medium to collect any remaining attached cells.
46. Transfer the rinse medium containing the remaining cells into the same 50 mL conical tube.
47. Add 2 volumes of N2B27 medium to stop the Accutase reaction.
48. Centrifuge the cells at 200 × *g* for 5 min at room temperature.
49. Resuspend the cell pellet in 5 mL N2B27 medium supplemented with Y-27632 at a 1:1000 dilution.
50. Gently dissociate cell clumps by pipetting with a 1000 μL pipette up to 5 times.

**CRITICAL:** Avoid excessive pipetting, as this may reduce cell viability and affect cell morphology after replating.

51. Count the cells.

**Pause point:** NCCs can be cryopreserved in Bambanker containing Y-27632 at a 1:1000 dilution. For optimal recovery,^5^ we recommend freezing the cells at a concentration of 3 × 10^6^ cells/mL.

52. Aspirate the laminin and poly-L-ornithine solution from the coated wells of 6-well plate.
53. Wash the coated wells twice with DPBS before plating the cells.
54. Add N2B27 medium supplemented with Y-27632 at a 1:1000 dilution, along with BDNF, GDNF, NT-3, and β-NGF each at 20 ng/mL to the culture plates.
55. Plate the NCCs at a density of 145,000 cells/cm^2^.

**CRITICAL:** The indicated plating density represents an empirically optimized target established through repeated testing of different seeding densities. Minor variations in cell density are generally well tolerated; however, substantial deviations should be avoided. Excessively low cell densities may reduce cell-cell interactions, attachment, survival, and differentiation efficiency, whereas excessively high densities may result in premature confluency, uneven differentiation, and increased non-neuronal cell overgrowth.

56. After 24 h, replace the medium with fresh N2B27 medium.

**CRITICAL:** Add medium slowly along the wall of each well. Do not dispense medium directly onto the plated cells, as this may detach newly attached sensory neurons. See troubleshooting problem 3.

**CRITICAL:** One day after replating, not all cells may have attached, and some cell debris may still be present. Therefore, perform the medium change carefully to avoid disturbing loosely attached cells. Conditioned medium was routinely preserved during all medium changes throughout the sensory neuron (SN) maturation phase. To retain secreted and cell-derived factors that may support neuronal survival and maturation, collect the existing medium, filter it to remove cellular debris, and combine it with the freshly prepared medium before adding it to the culture. Continue to monitor cell attachment and neurite outgrowth throughout the SN maturation phase. See troubleshooting problem 4.

57. On day 15 of sensory neuron differentiation, treat the cells with Mitomycin-C at a final concentration of 1 μg/mL for 2 h to reduce the non-neuronal cell population (Figure 3).

**Figure 3:**
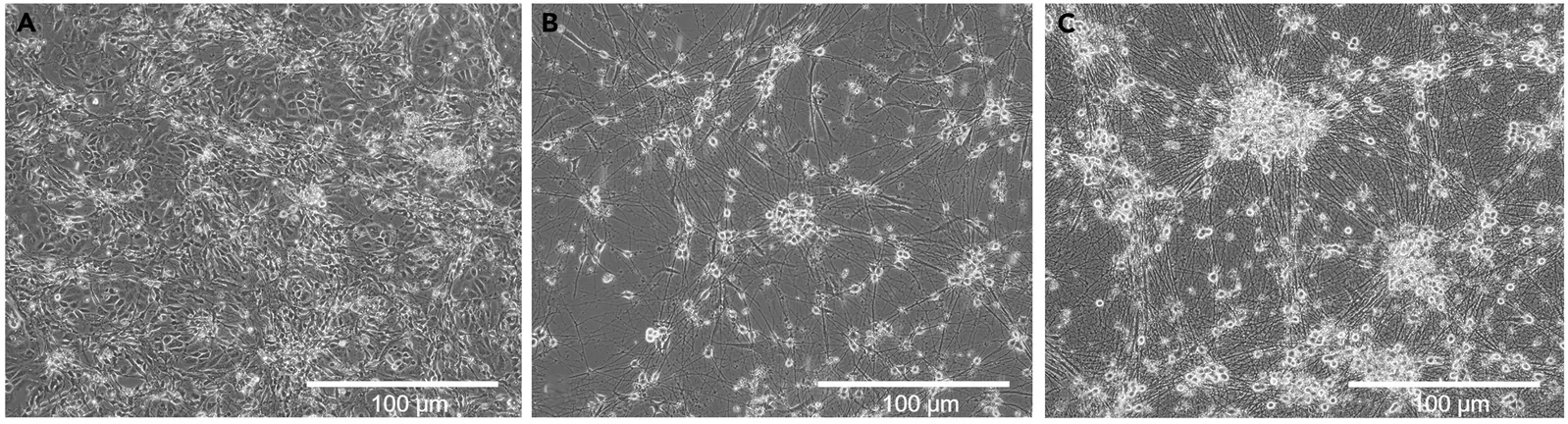
Effect of mitomycin-C treatment on iPSC-derived sensory neuron cultures Representative phase contrast images showing iPSC-derived sensory neuron cultures during mitomycin C treatment at DIV15. (A) Neuronal culture before mitomycin-C treatment. (B) Culture 24 h after mitomycin-C treatment. (C) Mature sensory neuron culture obtained following treatment and continued differentiation, showing successful neuronal enrichment and maturation. Scale bars, 100 μm.
58. After Mitomycin-C treatment, wash the cells with N2B27 medium. See troubleshooting problem 5.
59. Replace the medium with fresh N2B27 medium.
60. From day 15 onward, replace half of the medium twice per week until the end of differentiation (Figure 4).

**Figure 4:**
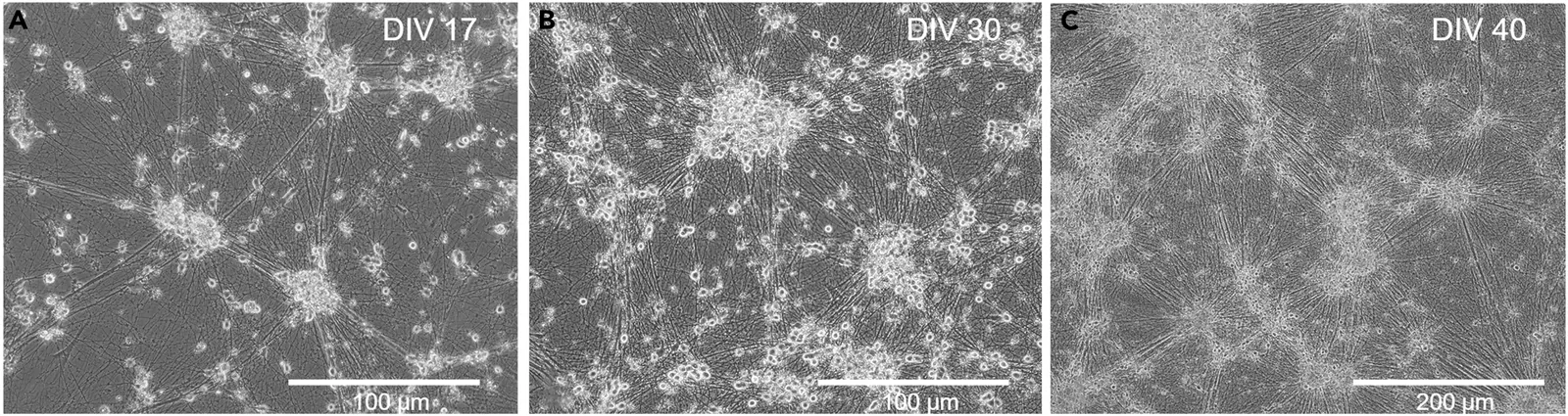
Phase images of iPSC-derived sensory neurons at different stages of maturation Representative phase contrast images showing the morphological progression of iPSC-derived sensory neurons during differentiation. (A) Premature neurons at DIV17, showing extended neurite outgrowth. (B) Sensory neurons at DIV30. (C) Sensory neurons at DIV40, showing a more mature and denser neuronal network. Scale bars, 100 μm in (A) and (B), and 200 μm in (C).

**CRITICAL:** Isolate RNA and protein samples at defined differentiation time points to monitor neuronal maturation and identify when the cultures are suitable for downstream experimental assays.

### Substance P release assay

**Timing: 3–4 h**

61. To stimulate substance P release, differentiated sensory neurons are treated with 1 mM and 10 mM capsaicin for 45 min. Untreated cell culture is maintained as the negative control, while 50 mM KCl stimulation is used as positive control.
62. Following the 45 min stimulation period, collect the cell-culture supernatants and centrifuge the samples at 1000 × *g* for 10 min to remove detached cells and debris.
63. Carefully transfer the supernatants into clean tubes and proceed immediately with the substance P ELISA.

***Note:*** Although the manufacturer permits storage of cell-culture supernatants in aliquots at −20^o^C or −80^o^C, however, all the samples used in this protocol are analyzed immediately after collection to avoid potential affects associated with sample storage or repeated freeze-thaw cycles.

64. For each sample well, add 40 μL of the supplied sample diluent followed by 10 μL of cell-culture supernatant. Perform all measurements in duplicate.
65. The resulting 1:5 sample dilution is accounted for by multiplying the concentration obtained from the standard curve by a dilution factor of 5.
66. Incubate the plate at 37^o^C for 40 min, wash the wells five times and, add 50 μL HRP-conjugate reagent followed by another 40-min incubation at 37^o^C.
67. After five additional washes, add 50 μL Chromogen Solution A and 50 μL Chromogen Solution B and incubate at 37^o^C for 20 min protected from light.
68. Add 50 μL of stop solution and immediately measure absorbance at 450 nm.
69. Determine the substance P concentration from the standard curve after blank subtraction and correct the values using the 5-fold dilution factor.

## Expected outcomes

This protocol is expected to generate neural crest cells (NCCs) from human iPSCs and subsequently differentiate them into sensory neurons (SNs) enriched for nociceptor-like cells. During NCC induction, cultures progressively undergo characteristic morphological changes, with aggregate and ridge-like structures becoming evident by day 8 and more clearly defined by day 11 (Figures 2A–2E). Successful NCC differentiation is supported by immunofluorescence staining for PAX6 and Nestin, together with qPCR analysis showing increased expression of NCC-associated markers at day 11 compared with day 8 (Figures 5A and 5B). Quantitative immunofluorescence analysis provides an additional objective criterion for assessing NCC induction, with 84.3% of cells being Nestin-positive and 80.9% being PAX6-positive (Figure 5C). Marker-positive cells are quantified relative to the total number of DAPI-stained nuclei. In addition, increased expression of Nestin, PAX6, SOX10, NGN1, and POU5F1 at day 11 indicates progression toward the NCC stage and supports the suitability of day 11 cultures for replating under sensory neuron differentiation conditions (Figure 5B).

**Figure 5:**
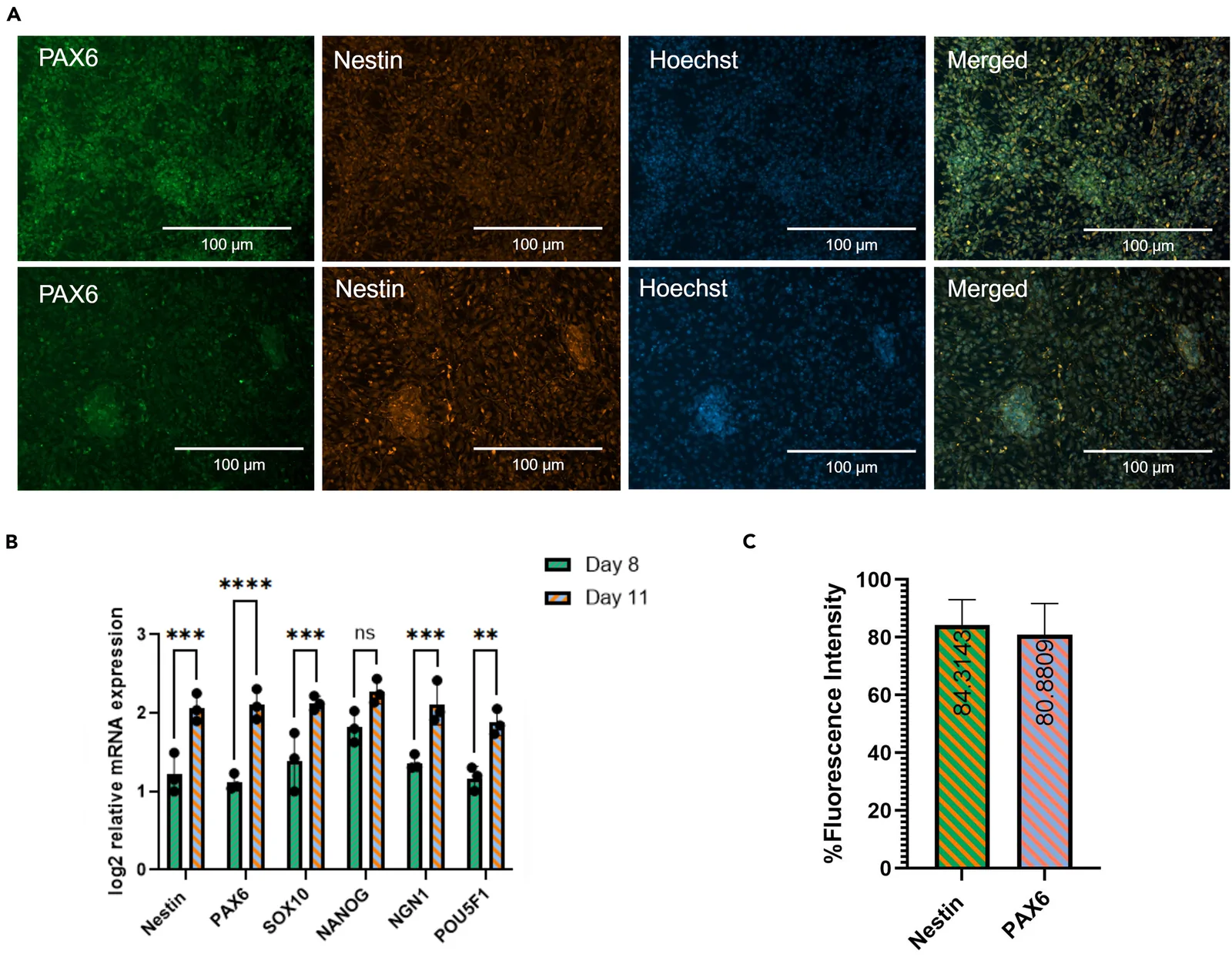
Characterization and validation of NCCs (A) Representative immunofluorescence images showing PAX6 and Nestin expression in differentiated neural crest cell cultures. Nuclei are counterstained with Hoechst, and merged images show marker expression together with nuclear staining. Scale bars, 100 μm. (B) RT-qPCR analysis of selected neural crest, neural, and pluripotency-associated genes at day 8 and day 11 of differentiation. Gene expression is normalized to ACTB and is shown as relative mRNA expression. Data are represented as Mean ± SEM. ∗p < 0.05, ∗∗p < 0.01, ∗∗∗p < 0.001, ∗∗∗∗p < 0.0001, one-way ANOVA, n = 3 (each n represents an independent differentiation). (C) Quantification of Nestin and PAX6 positive NCCs based on immunofluorescence staining. Bars represent the percentage fluorescence intensity for Nestin and PAX6, indicating robust expression of both NCC-associated markers in the differentiated cell population. Data are represented as mean ± SEM from n = 3 independent experiments.

Following replating, NCCs gradually mature into sensory neuronal cultures characterized by ganglion-like cell clusters and extensive neurite outgrowth (Figure 2A, DIV12). Treatment with Mitomycin-C at day 15 is used as a practical optimization to limit the overgrowth of proliferating non-neuronal cells and facilitate maintenance of an enriched neuronal culture. Mature SNs cultures show expression of the neuronal and peripheral sensory markers MAP2, Peripherin, and TRPV1 (Figure 6A). Quantitative immunofluorescence analysis shows that 74.4% of cells are MAP2-positive and 83.1% are Peripherin-positive, indicating that successful differentiations generate a predominantly neuronal population with a strong peripheral sensory neuronal phenotype and 84.3% of cells are TRPV1-positive, further supporting enrichment for nociceptor-like sensory neurons (Figure 6B). Western blot analysis additionally confirms expression of TRPV1, Peripherin, and ISLET1 in sensory neurons derived from both iPS12 and iMR90-4 cell lines (Figure 6F).

**Figure 6:**
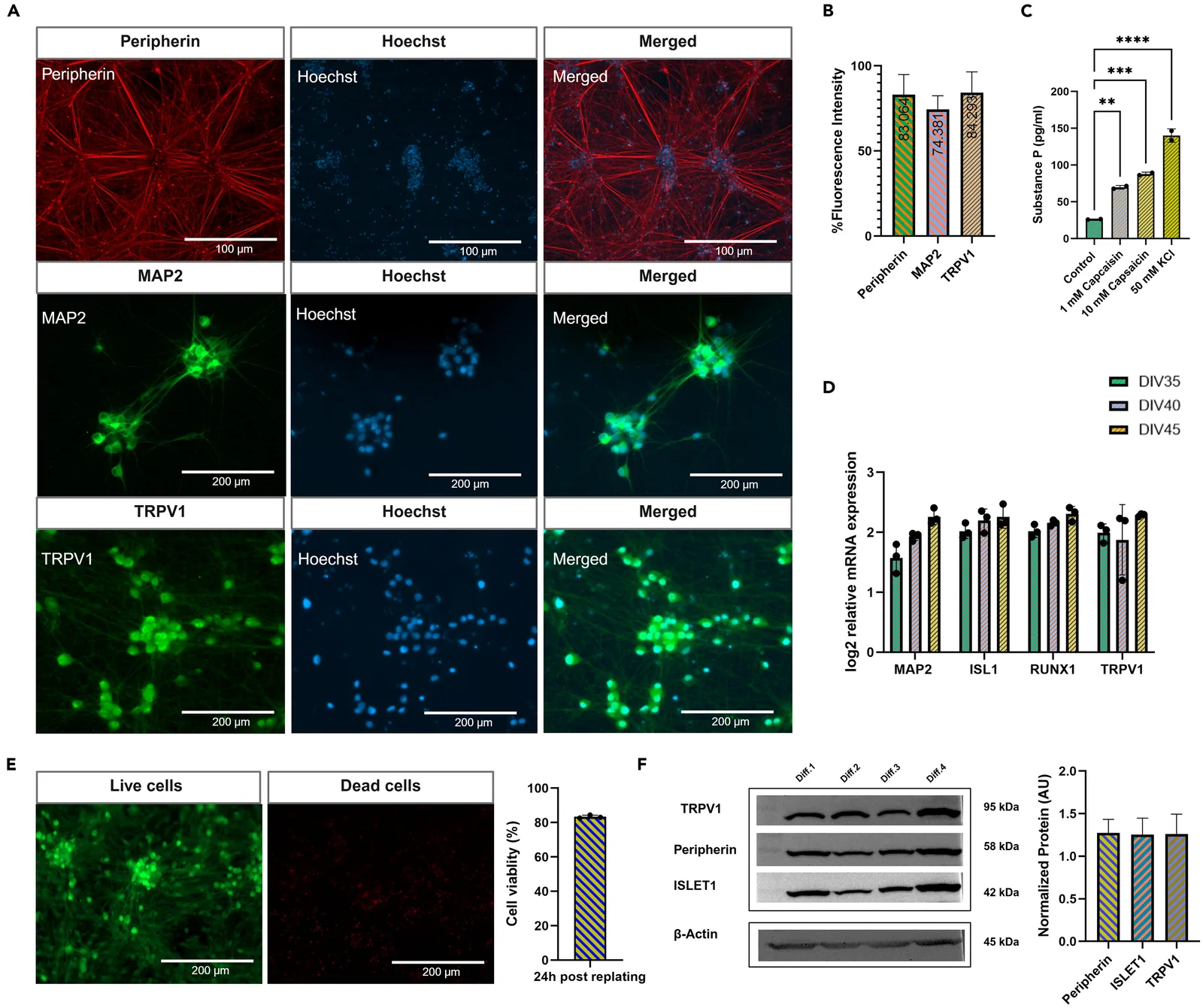
Characterization and functional validation of iPSC-derived sensory neurons (A) Representative immunofluorescence images showing expression of sensory neuron-associated markers Peripherin, MAP2, and TRPV1 in differentiated sensory neuron cultures. Nuclei are counterstained with Hoechst, and merged images show marker expression together with nuclear staining. Scale bars, 100 μm for Peripherin images and 200 μm for MAP2 and TRPV1 images. (B) Quantification of Peripherin, MAP2, and TRPV1 positive SNs based on immunofluorescence staining. Bars represent the percentage fluorescence intensity, indicating robust expression of SNs associated markers in the differentiated cell population. Data are represented as mean ± SEM from n = 3 independent experiments. (C) Substance P release measured in the supernatant of sensory neuron cultures following 45 min incubation at 37°C under control conditions or after stimulation with 1 mM capsaicin, 10 mM capsaicin, and 10 mMKCl. Data are represented as Mean ± SEM. ∗p < 0.05, ∗∗p < 0.01, ∗∗∗p < 0.001, ∗∗∗∗p < 0.0001, one-way ANOVA, n = 2 (each n represents an independent differentiation). (D) RT-qPCR analysis of sensory neuron-associated genes MAP2, ISL1, RUNX1, and TRPV1 at DIV35, DIV40, and DIV45. Gene expression was normalized to ACTB and is shown as log2 relative mRNA expression. Data are represented as Mean ± SEM from n = 3 independent differentiation experiments. (E) Representative fluorescence images showing live cells stained with calcein AM (green) and dead cells stained with BOBO-3 iodide (red) 24 h after replating. Quantification of cell viability, expressed as the percentage of viable cells at 24 h post-replating. Data are presented as mean ± SD. Scale bars from n=3, 200 μm. (F) Representative western blot showing ISLET1, Peripherin and TRPV1 expression in sensory neuron. β-actin was used as a loading control. Protein samples expression is normalized to β-actin. Data are represented as mean ± SEM from n = 3 independent experiments. Representative western blot data for sensory neurons markers is provided in Figure S1.

Cell viability following replating provides an additional quantitative indicator of culture quality. Live/Dead fluorescence analysis performed 24 h after replating shows a mean viability of 83.3% across independent experiments (Figure 6E). Under the conditions described here, a post-replating viability of approximately 80% or higher can therefore be used as a practical indicator of a healthy culture. Similarly, cultures showing approximately 80% positivity for major lineage-associated markers are generally representative of successful differentiation, although some variation between independent differentiations is expected.

The differentiated sensory neurons are expected to remain suitable for downstream experiments over an extended maturation period. qPCR analysis confirms continued expression of neuronal and sensory lineage markers, including MAP2, ISL1, RUNX1, and TRPV1, at DIV35, DIV40, and DIV45 (Figure 6D). Detection of these markers at DIV35 indicates that the cultures have reached a stage suitable for downstream experimental applications by this time point.

Functional maturation is further supported by stimulus-induced Substance P release into the culture supernatant. Compared with untreated control cultures, differentiated sensory neurons show increased Substance P release following capsaicin and KCl stimulation, with KCl producing the strongest response in the presented dataset (Figure 6C). Functional excitability is additionally assessed using Fluo-4 AM time-lapse calcium imaging. Following KCl-induced depolarization, differentiated iPSC-SNs exhibit a clear intracellular calcium response, demonstrating stimulus-evoked neuronal activity (Methods Video S1).

Methods Video S1. Fluo-4 AM time-lapse calcium imaging demonstrating neuronal activity in iPSC-derived sensory neurons

Together, the morphological, quantitative immunofluorescence, gene-expression, protein-level, biochemical, and calcium-imaging readouts support the successful generation of stable peripheral sensory neuronal cultures enriched for nociceptor-like cells. These cultures are suitable for downstream molecular, imaging, and functional applications from approximately DIV35 onward.

## Limitations

In our experience, a small proportion of differentiations may show suboptimal NCC ridge formation and reduced expression of NCC-associated markers, which can subsequently result in a lower sensory neuron yield (see troubleshooting 1). Occasionally, a limited number of Schwann-like cells may also be present in the final cultures and can be observed along the axonal projections of differentiated sensory neurons.

This protocol may require optimization depending on the iPSC line used. Differences in proliferation rate, attachment efficiency, and differentiation capacity between cell lines may require adjustment of the initial seeding density, replating density, or split ratio during differentiation. Therefore, each new iPSC line should be carefully monitored during the NCC induction and sensory neuron maturation stages, and minor protocol adjustments may be needed to obtain reproducible sensory neuron differentiation.

The differentiated cultures can be maintained for up to approximately 8 weeks under appropriate culture conditions. However, for reliable experimental use, we recommend performing downstream experiments within approximately 5 weeks, as neuronal morphology, viability, and functional stability are generally most consistent during this period.

An additional limitation is that the beneficial effect of Mitomycin-C treatment on reducing proliferating non-neuronal cells and improving neuronal purity is supported primarily by reproducible morphological observations and representative imaging. A direct quantitative comparison between Mitomycin-C treated and untreated cultures is not available; therefore, its effect should be considered a practical empirical optimization rather than a quantitatively validated improvement.

Although immunostaining, RT-qPCR, western blotting, Substance P release, and calcium imaging collectively support successful sensory neuronal differentiation and the acquisition of a nociceptor-like phenotype, these assays do not provide a complete functional characterization of mature nociceptor identity. Quantitative assessment of the proportion of capsaicin-responsive neurons and electrophysiological analyses, including action-potential firing and capsaicin-evoked currents, would provide additional functional validation. Furthermore, some validation assays are based on a limited number of biological replicates, including the Substance P release assay (n = 2), which limits statistical power and broad quantitative generalization. These data should therefore be interpreted as supportive functional validation within the broader multimodal characterization of the differentiated neurons. Future studies incorporating larger numbers of independent differentiations and more comprehensive functional analyses will further strengthen assessment of nociceptor identity, reproducibility, and protocol robustness.

## Troubleshooting

### Problem 1

NCC ridge formation is not observed, or the culture shows undefined cell types with irregular aggregate formation (Step 33, Figure 2B).

### Potential solution

Start NCC induction only from healthy iPSC cultures with clearly defined colony edges, minimal spontaneous differentiation, and the recommended confluency. Ensure that medium changes are performed daily and that all small molecules are added at the correct concentration and time point.

Use a lower passage number of iPSCs. We have found that maintaining the cells in culture for over 4–5 consecutive passages reduces the rate of successful differentiations.

### Problem 2

Incomplete single-cell dissociation after short Accutase exposure, followed by reduced cell viability due to excessive pipetting (Step 35).

### Potential solution

Allow Accutase to remain on the cells until the colonies dissociate into single cells without forceful mechanical disruption, it can even take 45 min. Monitor the cells under a microscope and proceed only when most cells have naturally detached and separated. After dissociation, gently pipette the suspension using a 5 mL pipette to obtain an even single-cell suspension while minimizing mechanical stress. To stop the Accutase reaction, add twice the volume of wash medium before centrifugation. This approach reduces excessive pipetting, preserves cell viability, and improves the quality of the single-cell suspension.

### Problem 3

Neurons detach during media change after replating (Step 48).

### Potential solution

Change the media very carefully during the first 24–48 h after NCC replating because newly attached cells are sensitive to mechanical disturbance. Aspirate slowly from the edge of the well and add fresh media along the wall instead of directly onto the cells. If needed, collect the existing conditioned media, filter it, and add it back with fresh media to support cell recovery.

### Problem 4

Poor neurite outgrowth during sensory neuron maturation (Step 48).

### Potential solution

Confirm that the laminin and poly-L-ornithine coating is freshly prepared and evenly distributed across the culture surface. Maintain the correct concentration of neurotrophic factors, including BDNF, GDNF, NT-3, and β-NGF, and avoid complete medium replacement during maturation. Poor coating quality, loss of trophic support, or excessive handling can reduce neurite extension and network formation.

### Problem 5

Cell death or reduced viability after Mitomycin-C treatment (Step 50).

### Potential solution

Treat SNs cultures with Mitomycin-C on day 15 rather than day 14, as the cells may still be stressed after replating on day 12. Limit Mitomycin-C exposure to 2 h to reduce toxicity, and wash the cells carefully after treatment. Before treatment, collect the conditioned medium and save it for recovery. After washing, replace the medium with a mixture of half fresh medium and half conditioned medium to support neuronal recovery, survival, and continued growth.

## Resource availability

### Lead contact

Requests for further information, resources, and reagents should be directed to and will be fulfilled by the lead contact, Ulf D. Kahlert (ulf.kahlert@med.ovgu.de).

### Technical contact

Technical questions on executing this protocol should be directed to and will be answered by the technical contact, Arslan Ghazanfar (arslan.ghazanfar@med.ovgu.de).

### Materials availability

This study did not generate new unique reagents.

### Data and code availability

The protocol includes neither datasets nor computer codes.

## Acknowledgments

This work was supported by grants from the EFRE program “PREDICT” (no. ZS/2023/12/186743). The authors gratefully acknowledge Dhanush Damodaran and Zain Ghazanfar for their helpful support and assistance during the preparation of this work. Illustrations in the figures were created using BioRender.com.

## Author contributions

Conceptualization, A.G. and U.D.K.; methodology, A.G., S.S., and M.I.S.; validation, A.G., J.P.K., and A.Y.S.; writing – original draft, A.G.; review and editing, U.D.K., U.S., C.H., and R.S.C.; supervision, U.D.K.; project administration, T.W.

## Declaration of interests

The authors declare no competing interests.
