# Supplementary material for: Protocol for generating nociceptor-enriched peripheral sensory neurons from human induced pluripotent stem cells
Source: STAR Protoc. 2026 Nov 4;7(4):104944. doi: 10.1016/j.xpro.2026.104944 (PMC13757024; doi:10.1016/j.xpro.2026.104944)
Supplement: Supplementary file 1 [file mmc1.pdf]

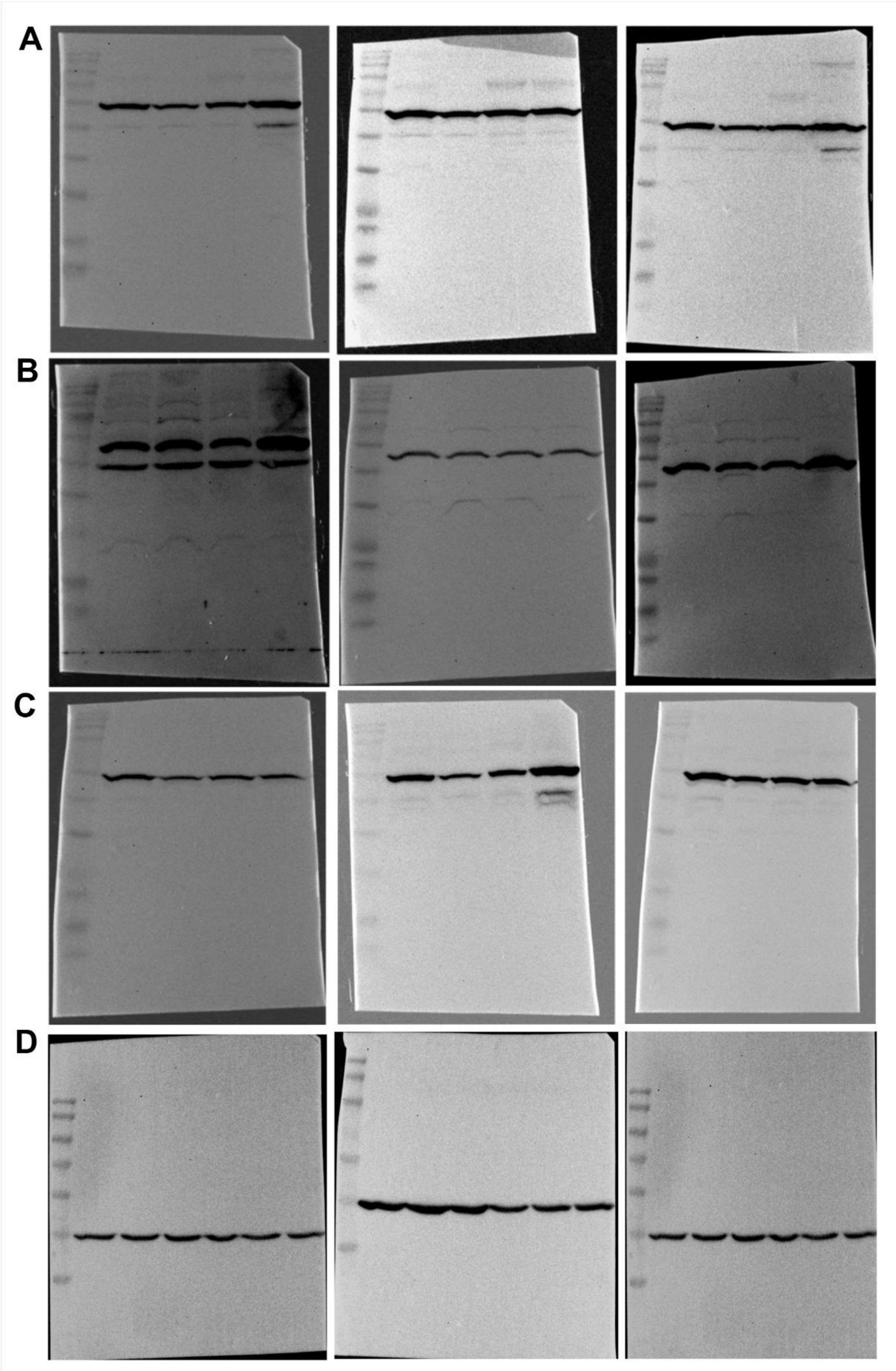

Figure S1. Western blot analysis of sensory neuron-associated proteins in hiPSC-derived sensory neurons.

Representative western blot images showing the expression of (A) Peripherin (58 kDa), (B) TRPV1 (95 kDa), and (C) ISLET1 (42 kDa) in hiPSC-derived sensory neurons. (D) β-actin (45 kDa) serves as a loading control, n=3.
